# Biomechanical Analysis of Various Connector Designs of Dental Implant Complex: A Numerical Finite Element Study

**DOI:** 10.1016/j.identj.2025.100873

**Published:** 2025-06-26

**Authors:** Shawkat A Elsheikh, Mohamed I El-Anwar, Tao Hong, Christoph Bourauel, Abdulaziz Alhotan, Noha M Anany, Islam G Shahin, Al-Hassan Diab, Tarek M Elshazly

**Affiliations:** a Faculty of Dentistry, Galala University, Suez, Egypt; b Implantology Department, Hospital of Stomatology, Xi'an Jiaotong University, Shaanxi, China; c Department of Mechanical Engineering, National Research Centre, Giza, Egypt; d Oral Technology, Dental School, University Hospital Bonn, Bonn, Germany; e Department of Dental Health, College of Applied Medical Sciences, King Saud University, Riyadh, Saudi Arabia; f Faculty of Dentistry, Ain Shams University, Cairo, Egypt; g Private Clinic, Abu Dhabi, UAE; h Department of Oral Medicine, Periodontology and Diagnosis, Faculty of Dentistry, British University in Egypt, Cairo, Egypt

**Keywords:** Biomechanics, Implant, Abutment, Bone, FEM, FEA

## Abstract

**Objective:**

To evaluate the biomechanical behavior of 5 types of commonly used implant/abutment connectors, using Finite Element Methods (FEM).

**Methods:**

Five models of implant-abutment connections were designed using computer-aided design (CAD) software: Tri-channel (M1), Conical internal hexagon (M2), Morse taper with an integrated screw (M3), Internal hexagon (M4), and Tube-in-tube (M5). The bone was modeled as coaxial cylinders, with the inner cylinder representing spongy bone and the outer 1 mm-thick cylinder representing cortical bone. A premolar crown geometry was designed onto the abutment with a 40 µm-thick cement layer. Three loading scenarios were applied to each model: (1) a 100 N compressive load, (2) a 50 N oblique load at 45° (relative to the implant axis), and (3) a 50 N lateral load.

**Results:**

All stress and deformation values remain within the tolerable limits for the materials used. Notably, M1, M4, and M5 exhibited optimal biomechanical performance. M1 and M4 recorded the lowest stresses in implant components, 24.4 to 24.8 MPa in the abutment and 27.5 to 27.9 MPa in the screw under compression, along with minimal crown deformation (8.6 µm compared to M3’s 11.7 µm). In contrast, M3 experienced the highest implant-component stresses (68.5 MPa in the abutment and 120.2 MPa in the screw) but showed the lowest cortical bone stress at 7.7 MPa, versus 10.2 MPa in M4.

**Conclusion:**

For long-term durability, implants with an internal hexagon (M4) or tri-channel (M1) design are preferable, as they minimize stress and deformation within the implant complex, thereby reducing the risk of prosthetic failure. While the Morse taper (M3) design may benefit patients with compromised bone density, its higher implant-component stresses warrant caution.

**Clinical significance:**

This study provides valuable insights to support evidence-based selection of implant–abutment connection designs. Among the 5 evaluated systems, the Tri-channel (M1) and Internal Hexagon (M4) designs demonstrated superior biomechanical performance by minimizing stress concentrations within the implant components and surrounding bone. These configurations are therefore recommended for routine clinical use to enhance prosthetic stability, reduce the likelihood of mechanical complications such as screw loosening or fracture, and prolong implant longevity. Conversely, although the Morse Taper with integrated screw (M3) design showed the lowest stress on cortical bone – suggesting potential benefit for patients with reduced bone quality – it exhibited the highest stress levels within implant components, indicating a higher mechanical failure risk. Clinicians should weigh these biomechanical trade-offs when planning treatment, particularly in patients with high functional loads or compromised bone conditions.

## Introduction

Dental implants are biocompatible, alloplastic materials surgically placed into the maxillary or mandibular bone to replace missing teeth lost due to congenital defects, trauma, or other causes.^1^^,^^2^ They are a reliable treatment option with a reported success rate of approximately 95 % over 5 years.^3^ Implant-supported prostheses are typically retained using either cement or screws. The choice of retention method and abutment type significantly impacts stress and strain within the system.^4^^,^^5^ The biomechanical performance of these prostheses is influenced by the distribution of stress and displacement within the implant complex, which includes the fixture, connector, and abutment. Excessive loading on these components can result in mechanical complications such as screw loosening, fractures, or abutment failure.^6^ Similarly, excessive loading on the surrounding bone can lead to bone resorption and may ultimately result in implant failure. Therefore, evaluating load distribution across different implant designs is essential to ensure long-term success.^7^, ^8^, ^9^

Stress-strain analysis employs diverse methodologies to evaluate internal forces (stresses) and deformations (strains) in materials and structures under external loads.^10^ In dental research, several techniques are commonly utilized for this purpose, including photoelastic analysis, digital image correlation, electrical resistance strain gauges, and the finite-element method (FEM).^11^^,^^12^ FEM provides detailed quantitative insights into the interactions between the prosthesis, implant, and adjacent bone.^13^^,^^14^ As a computational engineering tool for structural analysis, FEM is particularly effective for irregular geometries and heterogeneous materials. In FEM, the structure is divided into finite elements connected by nodes, and by using an appropriate mesh and proper mathematical models it can calculate the reactions and interactions within the structure. Moreover, when accurately configured with material properties, applied forces, and boundary conditions, FEM yields precise data on stress and strain distributions.^10^^,^^15^ This method can provide highly accurate predictions of potential shape changes and structural responses, offering valuable insights into the mechanical behavior and stability of the implant system.^11^^,^^13^^,^^16^

Dental implants are classified as 1-piece or 2-piece systems. One-piece implants are inserted and loaded in a single step but require sufficient bone for primary stability.^17^ Two-piece implants offer more flexibility: they can be immediately loaded or left to heal before a second-stage restoration, making them ideal for cases with limited bone or low bone density. Additionally, 2-piece implants allow for customized, angled abutments, enabling better adaptation to implant angulation and contributing to their broader clinical use and market popularity.^18^

The long-term success of 2-piece dental implants relies on a stable connection between the abutment and the implant.^19^ Various types of implant-abutment connections possess distinct mechanical and clinical characteristics, determining their performance and suitability for specific dental implant applications.^20^, ^21^, ^22^ The design of implant-abutment interfaces is categorized based on geometry and functional characteristics. External connections, like the external hexagon, have an interface located outside the implant body. Internal connections, such as the internal hexagon, internal conical, or Morse taper, position the interface inside the implant. While, external connections tend to have limited stability and lower durability due to their shallow connection and susceptibility to bending forces, internal connections allow for a longer and more secure interface and come in flat/cylindrical or conical designs.^23^ Hybrid connections combine both external and internal features to optimize their mechanical characteristics.^20^^,^^24^^,^^25^

In the current study FEM was utilized to evaluate the biomechanical performance of 5 common types of implant-abutment connectors, focusing on stress distribution and deformation within the implant complex. The aim was to provide numerical recommendations for selecting the most reliable connector design to improve the long-term clinical success and stability of dental implants, a topic that remains insufficiently addressed in the literature. The null hypothesis states that no substantial differences exist in biomechanical behavior among the 5 numerical designs.

## Methods

**Fig. 1:**
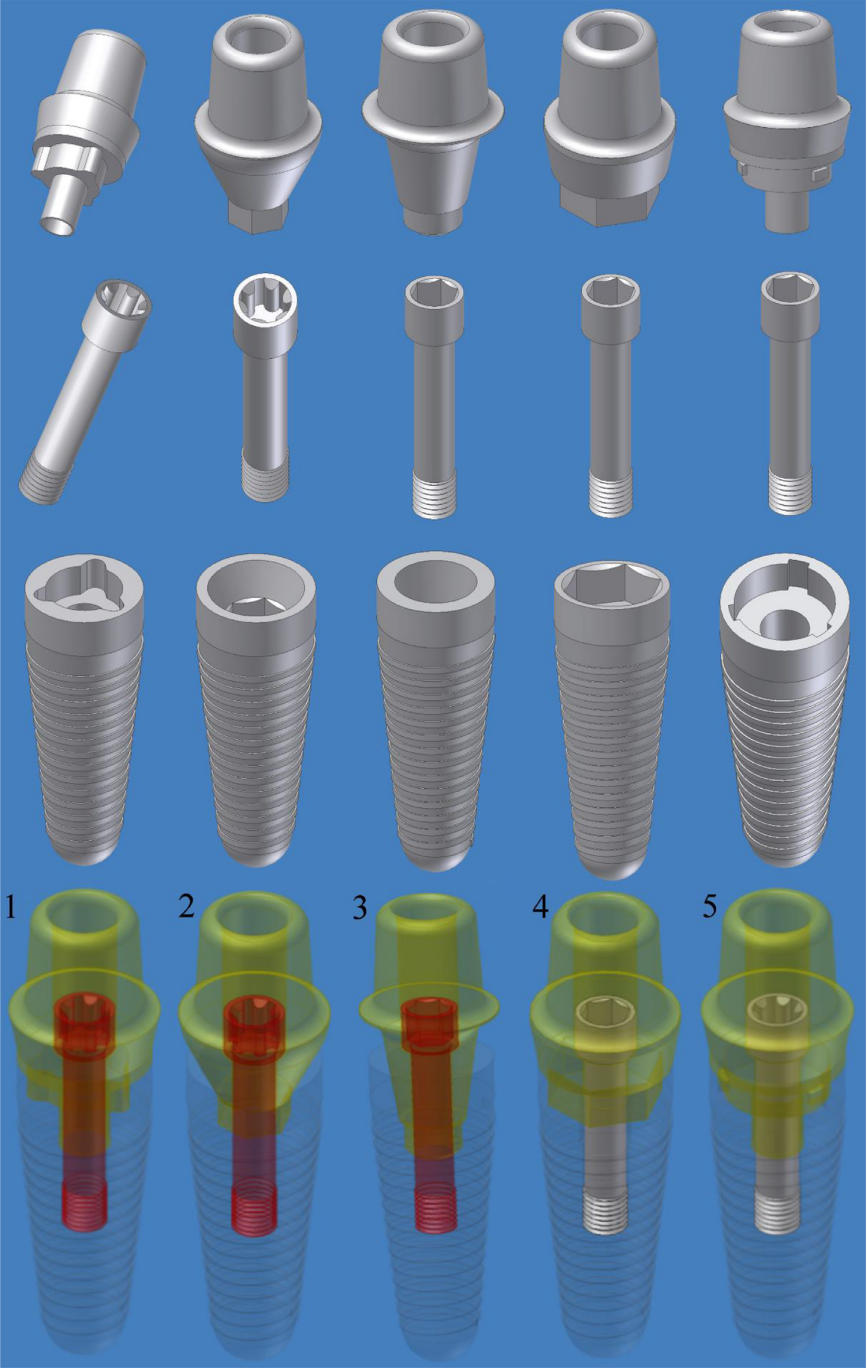
Illustration of different designs of implant-abutment connections: (1) Tri-channel; (2) Conical internal hexagonal; (3) Morse taper integrated screwed; (4) Internal hexagonal; and (5) Tube-in-tube implant-abutment connections.

Five models of implant-abutment connection were designed using AutoDesk Inventor CAD/CAM software (Autodesk, San Rafael, CA, USA), extracted from the manufacturers' catalogs as illustrated in Figure 1. These included: (M1) Tri-channel connection (Nobel Biocare, Kloten, Switzerland), (M2) Conical internal hexagonal connection (Nobel Biocare), (M3) Morse taper integrated screw-in connection (Neodent, Curitiba, Paraná, Brazil), (M4) Internal hexagonal connection (Implant Direct, LLC, Las Vegas, Nevada, United States), and (M5) Tube-in-tube connection (CAMLOG Biotechnologies, Basel, Switzerland). Additionally, the cortical and spongy bones were simplified and modeled as 2 coaxial cylinders. The spongy bone was represented by an inner cylinder measuring 14 mm in diameter and 22 mm in height, occupying the internal space of the outer cylinder, which had a 1 mm thick shell representing the cortical bone with dimensions of 16 mm in diameter and 24 mm in height.

**Fig. 2:**
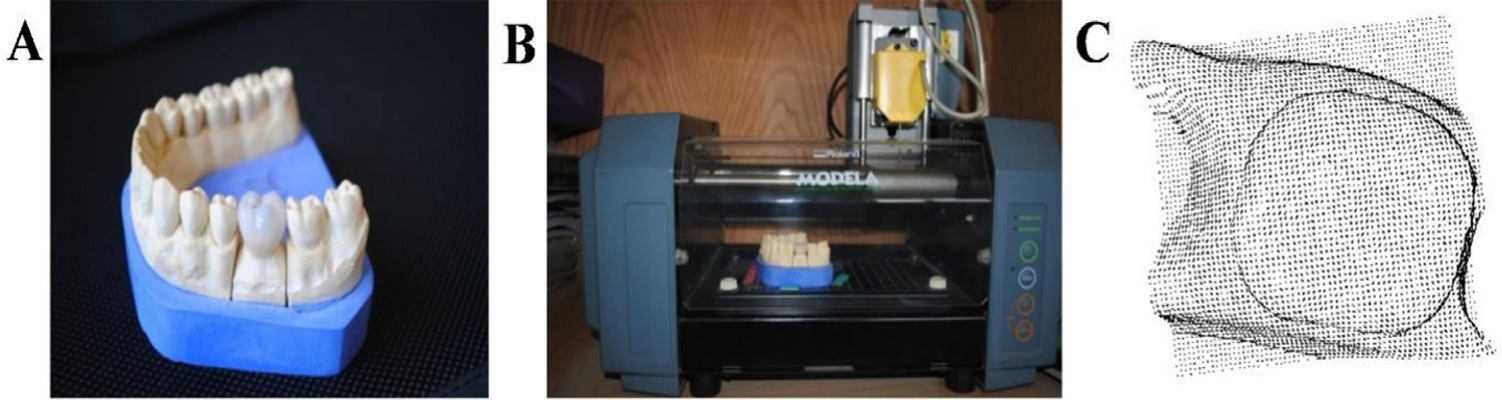
Steps involved in designing the digital model of the crown using a specialized 3D scanner: A, The physical crown specimen prepared for scanning, B, The Roland Modela contact probe scanner (model MDX-15) equipped with a Roland Active Piezoelectric Sensor used to capture the surface geometry, and C, The resulting cloud of points generated by the scanner represents the outer surface coordinates of the crown.

The outer surface of a suitable crown was scanned in 3D using a Roland Modela MDX-15 contact probe scanner (Roland DG, Tokyo, Japan), which uses a special piezoelectric sensor to capture precise details (Figure 2). The scanner’s software, Dr. PICZA 3, produced a digital file containing a cloud of points that mapped the crown’s shape. These points were then converted into a 3D mesh (a surface made of interconnected triangles) using Rhino software (Version 3.0, McNeel, Seattle, WA, USA). Next, the 3D model was trimmed horizontally along a flat plane to remove the lower portion of the crown, leaving only the top section for analysis. The cut edge at the bottom was smoothed and sealed to create a clean, closed shape.^26^ To model the cement layer (40 µm thick), the abutment (the structure beneath the crown) was digitally enlarged. The original abutment shape was then subtracted from this enlarged version using a Boolean operation, leaving a precise 40 µm gap to represent the cement space.

**Fig. 3:**
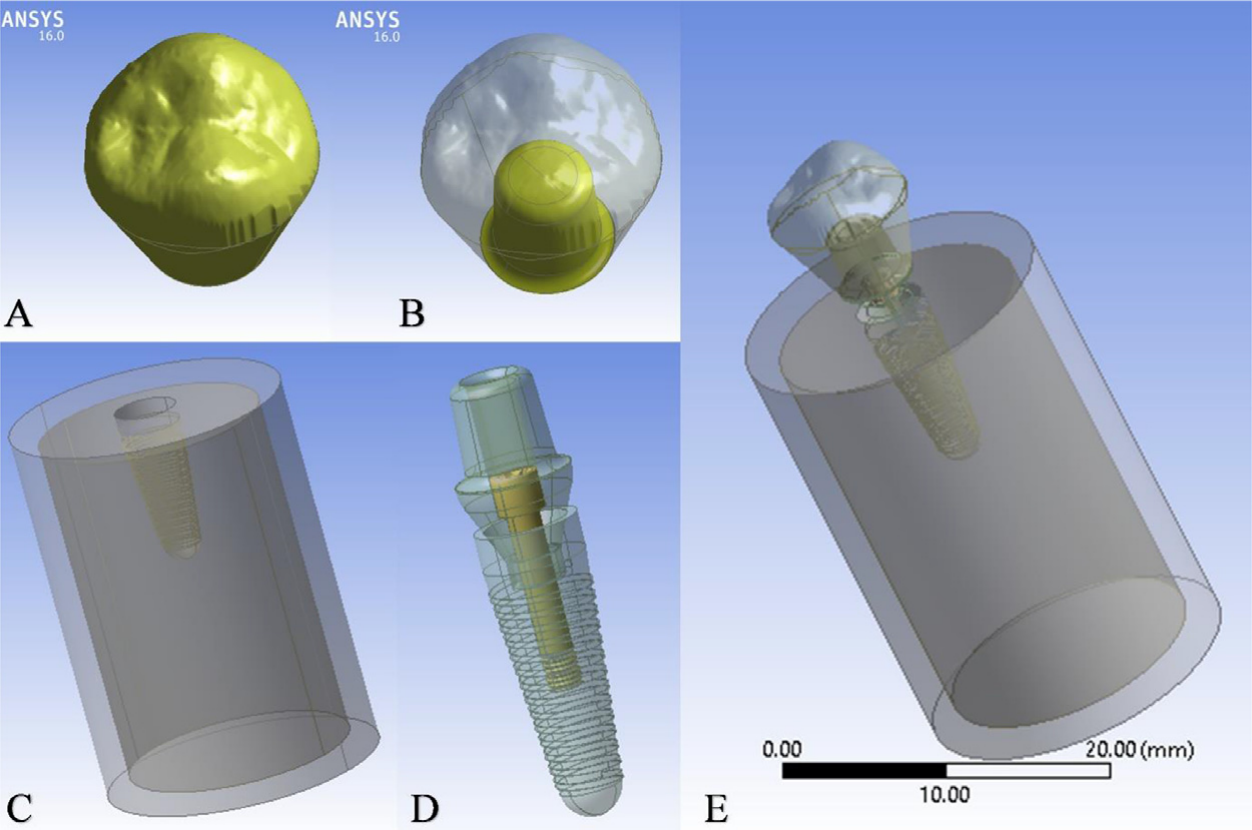
Components of the model: (A) Crown, (B) Crown and Cement, (C) Bone, and (D) Implants are complex (Abutments, Screws, Fixtures). (E) Components of the model: (A) Compact Bone, (B) Spongy Bone, and (C) Implant Fixture, (D) Three-units Prosthesis, (E) Implants are complex with the crown and cement.

The crown and implant models were assembled with the bone model and exported as Standard ACIS Text (SAT) files from the CAD software to finite element software (FE package ANSYS environment; ANSYS, Canonsburg, PA, USA) for further analysis. Boolean operations were utilized to establish precise contact surfaces between the system components (Figure 3). The solid geometries of the 5 models were discretized into finite elements using 3D hexahedral brick solid elements (Element Type 187). This element type has 3 translational degrees of freedom in the main global directions (XYZ).^27^ A mesh convergence test was performed to ensure accurate results by applying test loads at different mesh densities. This test determined the minimum number of elements needed for reliable results. The accuracy was verified by keeping the variation in von Mises stress on the cortical bone within ±5 %. The test showed that a smooth transition with a growth rate of 1.2 provided stable results, with element sizes ranging from 0.005 mm to 0.991 mm, capturing the details of the models effectively. The final mesh for each model included different numbers of elements and nodes, as shown in Table 1.

**Table 1:** Mesh density of the current models.

	M1		M2		M3		M4		M5	
	Nodes	Elements	Nodes	Elements	Nodes	Elements	Nodes	Elements	Nodes	Elements
Crown	303 268	217 812	303 268	217 812	303 268	217 812	303 268	217 812	303 268	217 812
Cement	21 586	10 352	21 586	10 352	21 586	10 352	21 586	10 352	21 586	10 352
Abutment	32 483	21 274	28 891	19 264	24 967	16 009	29 498	19 809	27 156	17 587
Screw	48 496	32 929	48 496	32 929	25 956	17 728	25 956	17 728	48 496	32 929
Implant	144 692	97 440	138 163	92 966	137 210	92 272	138 137	92 907	138 333	93 011
Cortical	71 349	43 812	71 349	43 812	71 349	43 812	71 349	43 812	71 349	43 812
Spongy	312 787	220 700	312 787	220 700	312 787	220 700	312 787	220 700	312 787	220 700

Glue contact was assigned to all interfaces between different components of each model, assuming full osseointegration between the bone and implant, as well as cold welding at the implant component interfaces.^28^ All materials were modeled as isotropic, homogeneous, and linearly elastic. The mechanical properties of the materials were assigned in ANSYS, as outlined in Table 2. The lower surface of the cortical bone was fixed in all 3 directions as a boundary condition to prevent movement during load application. Three loading conditions were applied to each model at the central fossa of the crown: (1) a compressive load of 100 N, (2) an oblique load of 50 N at a 45° angle, and (3) a lateral load of 50 N. Linear static analyses were conducted using a high-performance workstation (HP Z820; HP, Palo Alto, CA, USA) equipped with dual Intel Xeon E5-2660 processors (2.2 GHz) and 64 GB of RAM. The equivalent von Mises stress (EvM) and maximum total deformation in different model components are calculated.

**Table 2:** Mechanical properties of the materials used in the current finite element models.

	Modulus of elasticity in MPa	Poisson’s ratio	Reference
Crown: E-Max	95 000	0.26	Junior et al^29^
Cement: Rely X	7700	0.30	Shakir et al^30^
Titanium	110 000	0.25	De Moor et al^31^
Cortical Bone	13 700	0.30	Al-Zordk et al^32^
Spongy Bone	1370	0.30	Al-Zordk et al^32^

### Statistical analysis

FE simulations produce deterministic and consistent results for a given set of input parameters, as they operate based on predefined mathematical models and boundary conditions. Unlike experimental studies, which are influenced by variability and randomness, FE simulations yield fixed outputs for identical inputs. Consequently, statistical metrics such as mean and standard deviation, which rely on variability among data points, do not apply to FE studies. Instead, sensitivity analysis, as outlined earlier in the methodology, is more suitable for evaluating the impact of parameter variations in FE models.^10^

## Results

**Figure 4:**
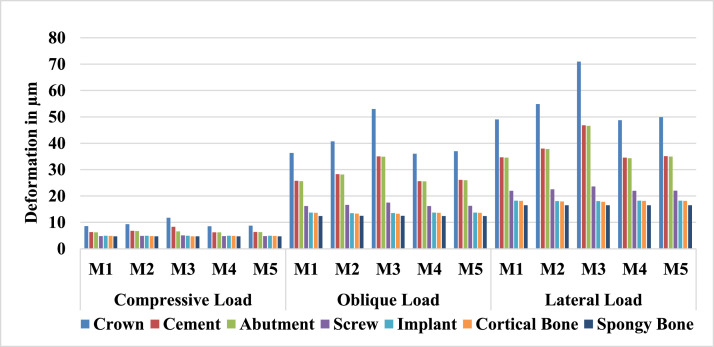
Maximum total deformation values for various components of the finite element models, comparing different designs of the fixture/abutment connector and different loading scenarios.

As shown in Figure 4, the deformation pattern of different objects is consistent across the 5 models, however, the deformation values vary drastically between objects and depending on the loading scenario or the model type. The deformation data reveal distinct biomechanical responses among the 5 implant connector designs (M1-M5) under compressive, oblique, and lateral loading. M3 consistently exhibited the highest deformation values across all components and loading scenarios, particularly in the crown (eg, 71.0 µm under lateral load vs 48.7-54.8 µm in other models) and cement (46.8 µm under lateral load vs 34.5-38.0 µm in others). In contrast, M1 and M4 demonstrated the lowest deformations, with near-identical values under compression (eg, crown: 8.6 µm) and lateral loads (eg, crown: 48.7 µm for M4 vs 71.0 µm for M3). Notably, deformation increased with load severity (compression < oblique < lateral), but critical components like the screw and implant showed minimal variation (eg, implant: 4.9 µm under compression across all models; 13.5-13.7 µm under oblique loads). Cortical and spongy bone deformation remained remarkably consistent (eg, cortical bone: 4.7-4.9 µm under compression; 17.8-18.2 µm under lateral loads), indicating that bone response is load-dependent but largely unaffected by connector design.

**Figure 5:**
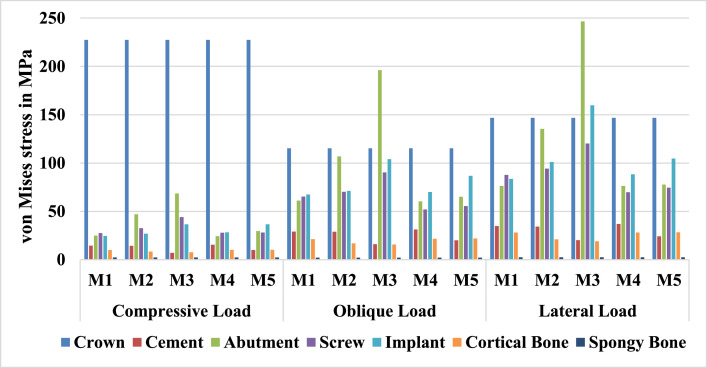
Equivalent von Mises stress results for various components of the finite element models, comparing different designs of the fixture/abutment connector and different loading scenarios.

In Figure 5, EvM values differ drastically between components of the models and are influenced by both the loading scenario and the type of implant-abutment connector. M3 consistently exhibited the highest stress concentrations in implant complex components (abutment, screw, and implant), reaching 68.5 MPa in abutment under compression and 120.2 MPa in screw under lateral load. In contrast, M1 and M4 demonstrated lower stresses in these components (M4 screw: 27.9 MPa under compression), indicating superior biomechanical stability of the prosthetic part. Notably, M3 redistributed stress away from cortical bone (7.7 MPa vs 10.2 MPa in M4 under compression), potentially reducing bone resorption, though at the cost of elevated implant stress. In the spongy bone, the differences in EvM values across models and loading scenarios were negligible and stayed at the lowest level compared to the other components. Cement stress varied with load type, with M3 showing the lowest compression-induced cement stress (7.1 MPa) but higher values under oblique and lateral loads. Crown stress remained uniform across designs but was load-dependent (reaching 227.5 MPa under compression).

**Figure 6:**
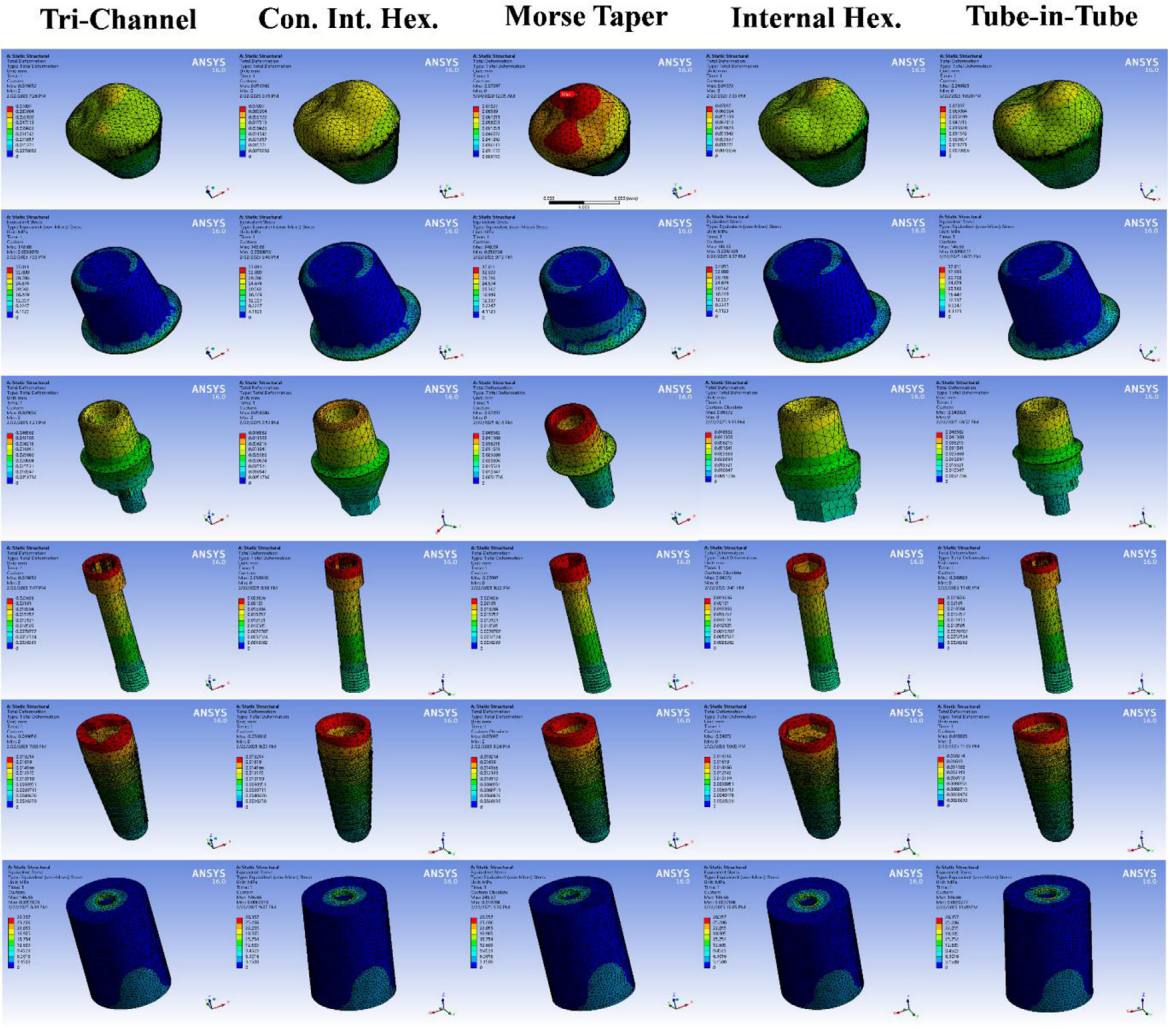
Color-coded screenshots of the lateral loading scenario showing the total deformation in the crown, abutment, connector screw, and implant, as well as the equivalent von Mises Stress distribution in the cement layer and cortical bone in the 5 different models from left to right: Tri-channel (M1); Conical internal hexagonal (M2); Morse taper integrated screwed (M3); Internal hexagonal (M4); and Tube-in-tube (M5) implant-abutment connections.

Figure 6 illustrates the total deformation and von Mises stress distribution across the 5 investigated models (M1-M5) under lateral loading. The cortical bone exhibited peak stresses at the implant-bone marginal interface, with M1 and M5 showing higher magnitudes than M2-M4, though stress patterns remained consistent. In contrast, the cement layer in M3 displayed a broader stress distribution compared to the localized marginal stresses in the other models. Crown deformation was greatest at the cusp tips, peaking in M3 and being minimized in M4. Similarly, deformation in the abutment, connector screw, and implant fixture concentrated at their coronal margins, with M3 demonstrating slightly higher displacement than others.

## Discussion

The loosening or fracture of abutment screws represents one of the most frequent mechanical complications in 2-piece dental implants, often resulting from micromovements and microgaps between the implant components.^33^ These mechanical issues can further trigger biological consequences, including bacterial infiltration at the implant–abutment interface and accelerated peri-implant bone loss.^34^ Consequently, reducing micromovements and microgaps is critical for ensuring the long-term stability of implants. To address this need, many manufacturers are already modifying implant geometries and incorporating improved anti-rotation mechanisms.^18^ In the present study FEA was employed to assess the biomechanical performance of 5 commonly used implant-abutment connection designs, focusing on stress distribution and deformation within the implant system, to provide evidence-based guidance for the selection of optimal connector designs, enhancing the long-term stability and clinical success of dental implants, a topic insufficiently researched in the literature.

The direction and magnitude of applied forces significantly influence stress patterns around dental implants.^35^ In the current FE model, 3 loading scenarios were applied to each finite element model to simulate clinically relevant conditions in the premolar and molar region. A 100 N vertical (axial) compressive load to represent normal occlusal forces during mastication.^36^ A 50 N oblique load at 45° to mimic functional forces during lateral chewing movements.^37^ A 50 N lateral load to simulate parafunctional activities such as bruxism.^38^

Calculated stress (MPa) and deformation (µm) values revealed notable differences among designs under specific loads, leading to rejection of the null hypothesis. Nevertheless, cortical bone stresses (7.7-10.2 MPa) fell safely below its typical compressive tolerance (100-200 MPa) and tensile tolerance (50-150 MPa).^39^ Moreover, deformation values (4.7-18.2 µm) also align with bone’s strain tolerance (<0.3% strain, ∼30 µm for a 10 mm section).^40^ As well, implant stresses (24.5-159.7 MPa) remained well within titanium’s strength limits (>500 MPa),^41^ indicating no risk of mechanical failure. Although short-term static loads – as simulated in the current study- suggest safety, long-term fatigue (cyclic loading) or patient-specific factors (eg, osteoporosis) -which are not considered in the current FE simulations- could alter risk profiles.^42^

The varying levels of deformation observed across the different models can be attributed to differences in their designs, which result in distinct mechanical behaviors.^5^ M3 (Morse Taper) and M2 (Conical Internal Hex) showed the highest crown deformation, which could negatively impact the chewing performance. The shared conical geometry in M2 and M3 appears to correlate with increased deformation and stress as the conical component size increases, likely due to minor sliding between the parts.^19^

Similarly, the total deformation of connecting screws showed minimal variation across models. However, M4 (internal hex) and M1 (Tri-channel) demonstrated superior and comparable performance, outperforming other designs. Elevated stress in M2 and M3 may stem from their conical connections, where minimal sliding against the interface walls concentrates stress on the screw. These findings align with Kanneganti et al,^43^ who observed higher screw damage in conical connections compared to Tri-channel designs due to uneven stress distribution. Similarly, Nokar et al^4^ reported that conical interfaces exhibited peak stresses at the abutment-screw junction and surrounding bone. Amornvit et al^44^ reported as well in FEA of finger prosthesis that the highest stress was at the head of the abutment screw, meaning the implant carried the most load. However, this stress is only 3.59% of the screw’s yield strength. The strongest part was the fixture collar.

The total deformation of the implant body (fixture) remained consistent across different models. However, stress distribution varied significantly, where M1 and M4 exhibited the lowest stress levels, with M1 reducing stress by approximately 40 % compared to M3. M3 demonstrated higher implant stress, likely due to conical connections forcing the abutment deeper into the implant cavity, concentrating stress at the fixture. Moreover, conical connections utilize a press-fit mechanism that creates strong physical contact and friction, potentially leading to cold welding.^13^^,^^28^ The resulting compressive forces enhance abutment adaptation, minimize the microgap, and allow the 2-piece system to function as a single cohesive unit. Although M5 (tube-in-tube) shares structural similarities with M1, its reduced contact area inherently amplifies stress, in agreement with Camps-Font et al,^45^ who reported elevated stress in tube-in-tube systems.

Although cortical bone deformations were similar across different implant–abutment connection types, von Mises stresses varied by as much as 30 %. Specifically, M3 exhibited the lowest cortical bone stresses, followed by M2, while the remaining 3 types performed comparably. These results align with previous studies,^46^, ^47^, ^48^ which reported that Morse taper connectors provide superior stress distribution in bone compared to external and internal hexagon designs. The improved performance is likely due to the conical abutment design that enables uniform load transfer to the implant and surrounding cortical bone, thereby reducing stress concentrations.

Our findings align with Camps-Font et al’s observations^45^ regarding the conical internal hex model’s ability to minimize bone–implant interface stress, but contrary to their report, we observed that Morse taper connections do not inherently induce higher bone stress compared to external hexagons or Tube-in-Tube designs, supporting Ceruso et al^20^ and Vinhas et al,^25^ internal connections (eg, internal conical, Morse taper) outperformed external hexagons in interface resistance, retention, and microgap reduction. Purely conical connections excel in sealing and bone preservation due to low bacterial leakage,^19^ but their lack of anti-rotational stability under torsional loads poses risks. While indexed designs improve rotation resistance, they compromise anti-bending strength,^22^ potentially leading to screw loosening and reduced de-torque values in angled abutment scenarios.^6^

The current FE models have some limitations, including the assumption of homogeneous, isotropic, linearly elastic material properties. These assumptions may not fully capture the anisotropic nature of biological structures like bone, which may lead to variations in predicted stress and strain patterns under more realistic conditions. Furthermore, the analysis utilized static loading, whereas physiological bone loading is inherently dynamic due to the dynamic manner of chewing, a discrepancy might affect the accuracy of tissue response predictions.^42^ Moreover, in the present study, only a single type of prosthetic material was utilized, although previous research^13^^,^^49^ has demonstrated that the choice of prosthetic materials significantly influences the biomechanical performance of the entire implant system. To address these limitations, future research should incorporate anisotropic and dynamic loading conditions to better mimic physiological bone behavior, integrate non-linear viscoelastic material properties for accuracy, include different prosthetic materials, and validate findings via clinical and experimental studies. Furthermore, fatigue simulations under cyclic loads and patient-specific modeling could predict long-term implant performance.

## Conclusions

Within the limitations of the study, the following could be concluded:

• For optimal crown and implant durability, prioritize M1 (Tri-channel) or M4 (Internal Hex) for their balanced stress distribution, while M5 (Tube-in-Tube) delivers moderate performance.
• M3 (Morse Taper) is outstanding in bone preservation and cement longevity, exhibiting the lowest stress levels in these areas. However, it poses a risk of implant-component failure due to elevated stresses on the screw and abutment.
• M2 (Conical Internal Hex) offers secondary advantages for bone health.

## Clinical recommendations

In patients with compromised bone density, M3 may be preferable, but careful monitoring of implant integrity is essential.

Ultimately, selecting a design depends on whether the priority is bone health or prosthetic longevity.

## Compliance with ethics requirements

This article does not contain any studies on human or animal subjects.

## Data availability statement

Data can be made available on request.

## Declaration of generative AI and AI-assisted technologies in the writing process

During the preparation of this work, the authors used the ChatGPT and DeepSeek AI tools to improve the readability of the English language. After using this tool, the authors reviewed and edited the content as needed and took full responsibility for the content of the publication.

## CRediT authorship contribution statement

*SE:* Methodology, Investigation, Data curation, Validation, Writing – review & editing. *ME:* Methodology, Investigation, Data curation, Validation, Writing – review & editing. *TH:* Methodology, Investigation, Data curation, Validation, Writing – original draft, Writing – review & editing. *CB:* Resources, Writing – review & editing. *AA:* Writing – review & editing. *NA:* Writing – review & editing. *IS:* Writing – review & editing. *AD:* Writing – review & editing. *TE:* Data curation, Validation, Resources, Writing – original draft, Writing – review & editing. All authors have read and agreed to the published version of the manuscript.

## Conflict of interest

None disclosed.
